# Strengthening data collection for neglected tropical diseases: What data are needed for models to better inform tailored intervention programmes?

**DOI:** 10.1371/journal.pntd.0009351

**Published:** 2021-05-13

**Authors:** Jaspreet Toor, Jonathan I. D. Hamley, Claudio Fronterre, María Soledad Castaño, Lloyd A. C. Chapman, Luc E. Coffeng, Federica Giardina, Thomas M. Lietman, Edwin Michael, Amy Pinsent, Epke A. Le Rutte, T. Déirdre Hollingsworth

**Affiliations:** 1 Big Data Institute, Li Ka Shing Centre for Health Information and Discovery, Oxford, United Kingdom; 2 London Centre for Neglected Tropical Disease Research, Department of Infectious Disease Epidemiology, Imperial College London, London, United Kingdom; 3 Medical Research Council Centre for Global Infectious Disease Analysis, Department of Infectious Disease Epidemiology, School of Public Health, Imperial College London, London, United Kingdom; 4 Centre for Health Informatics, Computing and Statistics, Lancaster University, Lancaster, United Kingdom; 5 Department of Epidemiology and Public Health, Swiss Tropical and Public Health Institute, Basel, Switzerland; 6 University of Basel, Basel, Switzerland; 7 Department of Global Health and Development, London School of Hygiene and Tropical Medicine, United Kingdom; 8 Department of Medicine, University of California, San Francisco, California, United States of America; 9 Department of Public Health, Erasmus MC, University Medical Center Rotterdam, Rotterdam, the Netherlands; 10 Francis I Proctor Foundation, University of California, San Francisco, California, United States of America; 11 Department of Ophthalmology, University of California, San Francisco, California, United States of America; 12 Department of Epidemiology & Biostatistics, University of California, San Francisco, California, United States of America; 13 Department of Biological Sciences, University of Notre Dame, Notre Dame, Indiana, United States of America; 14 Department of Infectious Disease Epidemiology, London School of Hygiene & Tropical Medicine, London, United Kingdom; University of Heidelberg, GERMANY

## Abstract

Locally tailored interventions for neglected tropical diseases (NTDs) are becoming increasingly important for ensuring that the World Health Organization (WHO) goals for control and elimination are reached. Mathematical models, such as those developed by the NTD Modelling Consortium, are able to offer recommendations on interventions but remain constrained by the data currently available. Data collection for NTDs needs to be strengthened as better data are required to indirectly inform transmission in an area. Addressing specific data needs will improve our modelling recommendations, enabling more accurate tailoring of interventions and assessment of their progress. In this collection, we discuss the data needs for several NTDs, specifically gambiense human African trypanosomiasis, lymphatic filariasis, onchocerciasis, schistosomiasis, soil-transmitted helminths (STH), trachoma, and visceral leishmaniasis. Similarities in the data needs for these NTDs highlight the potential for integration across these diseases and where possible, a wider spectrum of diseases.

## Introduction

The neglected tropical diseases (NTDs) are a diverse group of communicable diseases identified by the World Health Organization (WHO) which predominantly affect populations living in poverty, leading to increased morbidity and mortality [1]. In 2012, WHO Roadmap on NTDs was developed to accelerate efforts for elimination and control whereby the diseases are no longer considered public health problems [1]. Disease-specific goals have been defined and set by WHO to be reached by 2020 with new Roadmap targets drafted for 2021 to 2030 [2]. High-quality data are needed to track progress towards the new WHO NTD Roadmap, but data challenges remain [3]. Furthermore, WHO recognises that monitoring and evaluation (M&E) for all NTDs is weak in many countries and that the capacity for data collection should be prioritized and strengthened [2].

Moving forward, it is clear that there is a need to strengthen data collection and evaluation for decision-making. Mathematical models, such as those developed and investigated by the NTD Modelling Consortium [4–6], have an important role in evaluating current data and determining remaining data gaps. These models have recently been recognised by WHO for providing information to inform strategies against NTDs [7,8].

To inform the discussion on expanding data collection, we have performed focused analyses on priority data needs for 7 NTDs (gambiense human African trypanosomiasis, lymphatic filariasis, onchocerciasis, schistosomiasis, soil-transmitted helminths (STH), trachoma, and visceral leishmaniasis in the Indian subcontinent) in a special collection of papers in *PLOS Neglected Tropical Diseases* and summarised the key data requirements raised within this special NTD Modelling Consortium collection here [9]. These analyses address 2 main issues: Firstly, M&E needs to better inform tailoring of programmes, and secondly, key epidemiological uncertainties which are crucial for understanding the dynamics of these diseases in response to interventions and in planning for WHO control or elimination goals.

Although this collection was written prior to the current Coronavirus Disease 2019 (COVID-19) pandemic which has postponed many NTD-related activities [10], upon their resumption, there is an opportunity to collect data which could be used to better tailor programmes, ensuring and, in some cases, accelerating progress towards WHO 2030 targets [11].

## Indirectly estimating transmission

To reach WHO goals by 2030, tailoring of intervention programmes is becoming increasingly important, particularly as many of the NTDs face programmatic constraints (Table 1). Measures of transmission in an area are required to inform model-based recommendations for tailored interventions, i.e., the frequency, coverage, and duration of interventions required. However, as disease transmission cannot be directly measured, it must be estimated indirectly from data collected in the field. In most areas, local tailoring of interventions requires more information on local transmission than current surveillance delivers.

**Table 1:** Overview of the 7 NTDs analysed in the NTD Modelling Consortium collection [9].

NTD and WHO target analysed in collection	Main mode of transmission	WHO-recommended strategy
Gambiense human African trypanosomiasis: Elimination of transmission [12]	Transmitted by tsetse flies	Intensified disease management via active and passive case finding, followed by treatment
Lymphatic filariasis (Elephantiasis): Elimination as a public health problem (<1% microfilarial prevalence) [13]	Transmitted by mosquitoes	Annual MDA
Onchocerciasis (River blindness): Elimination of transmission [14–15]	Transmitted by black flies	Annual MDA
Schistosomiasis (Bilharzia): Morbidity control (≤5% heavy-intensity prevalence in school-aged children aged 5–14 years) and elimination as a public health problem (≤1% heavy-intensity prevalence in school-aged children aged 5–14 years) [16]	Transmitted through parasite eggs in an infected individual’s excreta contaminating freshwater sources. Cercariae, then released by freshwater snails, penetrate the skin infecting individuals during contact with the water	MDA once every 3 years/2 years/1 year (frequency determined by the prevalence of infection in school-age children)
STH (Intestinal helminths): Elimination as a public health problem (≤2% moderate-to-high intensity prevalence in school-aged children aged 5–14 years) [17–18]	Transmitted through helminth eggs in feces contaminating the environment (soil). Individuals are infected through the ingestion of eggs (*Ascaris* and *Trichuris*) or by walking barefoot on contaminated soil (hookworm) as larvae penetrate the skin	MDA once every 2 years/1 year (frequency determined by the prevalence of infection in school-age children)
Trachoma: Elimination as a public health problem (<5% prevalence of follicular trachoma in children aged 1–9 years) [19–20]	Transmitted through an uncertain combination of vectors and direct contact	Annual MDA
VL: <1 new VL case per 10,000 population per year at subdistrict level (India and Bangladesh)/district level (Nepal) [21–23]	Transmitted by sandflies	Twice yearly indoor residual spraying of insecticide and active case detection followed by (free) VL treatment

MDA, also referred to as preventive chemotherapy, is a large-scale periodic treatment with treatment drugs.

MDA, mass drug administration; NTD, neglected tropical disease; STH, soil-transmitted helminths; VL, Visceral leishmaniasis; WHO, World Health Organization.

**Fig 1:**
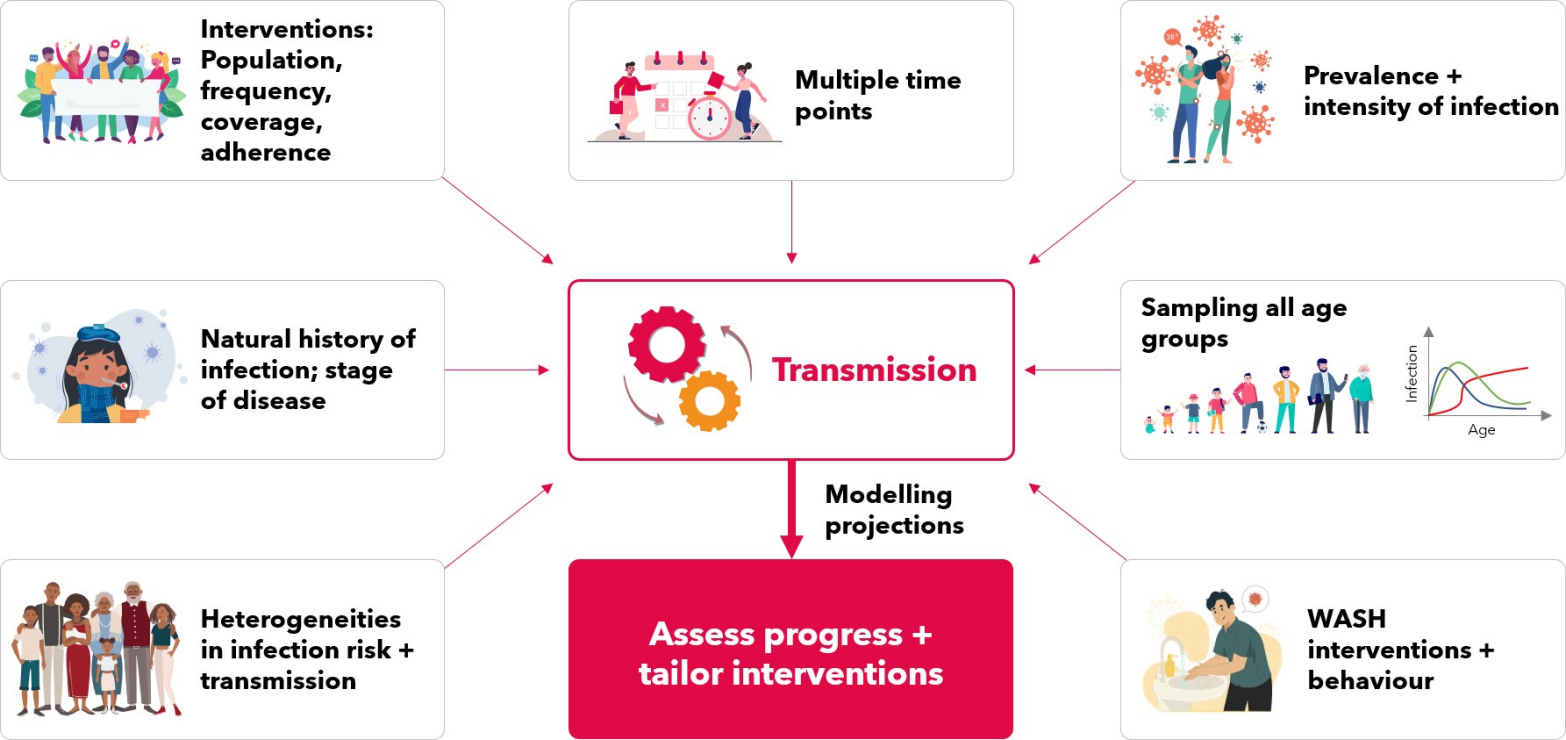
Key data required to indirectly inform transmission which feeds into and improves modelling projections allowing for better assessment and tailoring of interventions. WASH, water, sanitation, and hygiene.

Mathematical models have the potential to offer recommendations for locally tailored interventions but remain constrained by the data currently available. Better data will improve the quality of models and modelling recommendations in numerous ways, such as informing model parameters and assumptions, reducing uncertainty and verifying projections, thereby enabling more accurate tailoring of interventions and assessment of their progress. There are many ways to improve data collection activities to gain more information about transmission (summarised in Fig 1 and Tables 2 and 3).

**Table 2:** Summary of M&E data needs for 6 NTDs.

NTD	M&E quantity	Why is it important to collect this?	How could this be measured?	Constraints and caveats
**Infection data at various time points from all age groups**				
**Lymphatic filariasis [13]**	Human infection (mf and CFA prevalence) and mosquito abundance surveillance data at baseline, pre-and post-TAS from representative/sentinel monitoring sites. MDA, any vector control type and coverage data on these sites Sequential field studies to assess infection absence in mosquito samples	To validate model predictions, estimate breakpoints, obtain better diagnostic tool performance statistics, and facilitate model-data based area-wide freedom from infection calculations. To determine efficient spatially explicit intervention strategies and remedial options	Sampling of infection status from all age groups. Vector abundance data could be surveyed from spatially representative sites using appropriate traps or assembled from corresponding malaria programmes Develop and use sequential mosquito sampling protocols to evaluate interruption of transmission	Constraints with current data sharing protocols. ESPEN data not detailed enough. Difficult to obtain samples from adults and mosquito abundance data. Diagnostic tool performance statistics still undetermined
**Schistosomiasis [16]**	Broader age-intensity of infection data	To inform the age profile of infection and to determine settings where adults need to be sampled in addition to SAC. To determine the optimal treatment strategy, i.e., whether adults need to be treated in addition to SAC and at what coverage levels	Sampling from all age groups to collect intensity data, particularly SAC and adults (at least at baseline)	Difficult to obtain samples from adults. Limited drug donations for adults. Diagnostic tools less sensitive as prevalence and intensity decline
**VL in the Indian subcontinent [21]**	Repeated cross-sectional (or longitudinal) serological and LST measurements	To determine whether infection rate is age dependent and to model asymptomatic infection dynamics more accurately. To determine which interventions will have the biggest impact. To determine whether serological assays can be used to monitor progress towards elimination	Sampling from all age groups and running quantitative serological assays with consistent standardisation	Not feasible to take blood samples and run serological assays at population scale except in research settings. A species-specific LST antigen is not currently produced under good manufacturing practices
**Trachoma [19]**	Sero-positivity status within the same population Longitudinal individual level data on infection and TF prevalence	To help inform sero-reversion and/or antibody decay rates. To validate model predictions	Repeated cross-sectional surveys before, during, and after MDA. TF surveillance and PCR testing of individuals over time as prevalence declines	Surveillance is costly. Not many communities are completely treatment naïve Requires its own individual study. Hard to find areas with declining prevalence that could be monitored over a long period
**Natural history of infection**				
**Gambiense human African trypanosomiasis [12]**	Stage of disease of reported cases	To better capture improvements in passive case detection and to reduce uncertainty in estimates of subsequent reduction in transmission	Staging is part of routine screening protocols but staged data are not systematically recorded	Staging information may no longer be collected if new diagnostic tools and treatments are stage independent
**Heterogeneities in infection risk and transmission**				
**STH [17]**	Prevalence distribution in each IU	To assess whether the morbidity goal has been met in an IU and to determine the treatment frequency required	Sampling SAC in a higher number of villages/schools per IU	Logistics and costs associated with increasing the number of sentinel sites (schools)

CFA, circulating filarial antigens; ESPEN, expanded special project for elimination of NTDs; IU, implementation unit; LST, leishmanin skin test; MDA, mass drug administration; M&E, monitoring and evaluation; mf, microfilaraemia; NTD, neglected tropical disease; PCR, polymerase chain reaction; SAC, school-aged children; STH, soil-transmitted helminths; TAS, transmission assessment survey; TF, trachomatous follicular inflammation; VL, Visceral leishmaniasis.

**Table 3:** Summary of epidemiological data needs for 4 NTDs.

NTD	Epidemiological quantity	Why is it important to collect this?	How could this be measured?	Constraints and caveats
**Heterogeneities in infection risk and transmission**				
**Onchocerciasis [14]**	Human/blackfly mixing patterns based on pre-control distribution of mf intensity levels in humans Mean larval infection intensity per local blackfly population and the size of potential human subgroups linked to the same sites (e.g., fishermen near a specific fly-breeding site)	Model-predicted prospects of elimination through MDA strongly depend on the degree of assortative mixing. However, there is little quantitative evidence to inform elimination strategies on whether and how to respond to assortative mixing	Sampling from diverse individuals (skin snips). In settings with mf prevalence <30%, high skin mf density in those mf-positive (>20 mf/skin snip) may indicate assortative mixing Interviewing the local human population (asking for main visited locations) and catching and dissecting blackflies from diverse locations. Trying to link local fly populations with high infection intensity levels to specific human subgroup(s) exposed to these flies	Difficult to quantify the extent of assortative mixing. Highly location-specific data and entomologist expertise are needed
**Onchocerciasis [15]**	Individual-level heterogeneity in exposure to fly bites	Exposure heterogeneity has a large impact on parasite resilience and is currently estimated using population level epidemiological data	Development of anti-saliva antibody assays for simuliids (similar work done on Leishmania infantum transmission in dogs)	Heterogeneity in susceptibility may also be expected but it is not clear how to account for or estimate this in the current model in the context of the proposed data collection
**VL in the Indian subcontinent [22]**	GPS locations of VL cases/non-cases, sandfly density, and infection prevalence	To better understand the sources of spatial clustering (how this varies with endemicity, sandfly density and infection) and better predict village-level incidence. To improve targeting of interventions in space and time	Cross-sectional surveys of endemic communities recording household locations and trapping flies with light traps to test them for infection	Recording GPS data for all individuals and trapping and testing sandflies is highly resource intensive and only feasible in limited research settings
**Trachoma [20]**	Rate of return (growth or decay) of infection post MDA and efficacy of azithromycin in reducing infection in an individual and a population	While many parameters are unknown, knowledge of these two alone allow forecasting with different strategies	Repeated measurement of infection by PCR	Heterogeneity across regions. Only a few programmes are experienced monitoring infection
**Natural history of infection**				
**VL in the Indian subcontinent [23]**	Immune status of individuals (preferably longitudinal >15 years) and the prevalence of ongoing infection, including asymptomatic infections	Duration of immunity is important when simulating resurgence risks post-elimination. Markers for infection need to be identified	Humoral immune response to be tested with DAT and cellular immune response with LST. DAT titres and rK39 antibody levels combined with presence/absence of VL symptoms as markers for infection	The availability of LST. Continuation of existing projects is essential for longitudinal data. Data on asymptomatic infectiousness from recent xenodiagnosis studies have just become available [24]
**WASH interventions and behaviour**				
**STH [18]**	Potential correlation between uptake of WASH interventions and pre-WASH infection levels. Load and survival of eggs in the environment before and during WASH	Disentangle the impact of WASH interventions that reduce environmental contamination from those that reduce exposure to the environmental reservoir of infection. To better understand and predict the value of WASH and to determine WASH uptake levels needed to scale down PC	Detailed observation and documentation of WASH-related behaviour	WASH-related behaviour is difficult and expensive to measure and quantify. Low reliability of self-reported WASH-related behaviour. Lack of standardised and reproducible method of measuring environmental contamination

DAT, direct agglutination test; LST, leishmanin skin test; MDA, mass drug administration; M&E, monitoring and evaluation; mf, microfilaraemia; NTD, neglected tropical disease; PC, preventive chemotherapy; PCR, polymerase chain reaction; STH, soil-transmitted helminths; VL, Visceral leishmaniasis; WASH, water, sanitation, and hygiene.

## Improving monitoring and evaluation

To improve the outcomes and impact of NTD interventions, M&E activities are carried out to enhance performance and measure results [2]. A vital aspect of M&E is collecting data which can be used to assess whether interventions are on track for achieving WHO goals. To assess this and to determine areas where interventions need to be modified (e.g., intensified due to not being on track or relaxed due to being overtreated/limited resources), more information about the interventions being implemented is needed. This includes data on the population that has been targeted, the timing and frequency of interventions, and additionally for mass drug administration (MDA) programmes, the coverage and adherence during each round of MDA (Fig 1).

M&E data can be used to determine the optimal treatment strategy (i.e., frequency, coverage, and duration) required in a particular location (Table 2 and Fig 1). To determine the specific age groups that need to be targeted in a given area, data are required to inform the age profile of infection [13,16,21].

To assess how infection levels are impacted following a round of treatment, and to validate model projections, data collected at multiple time points, particularly pre- and posttreatment, are informative [13,16,19]. Furthermore, for diseases assessing the effectiveness of passive case detection, such as gambiense human African trypanosomiasis, data on the stage of the disease are needed [12]. Where possible, collecting data at multiple time points within randomised controlled trials can provide greater insight into the impact attributable to an intervention.

It is important to note that reality cannot be perfectly observed but collecting better data and using statistical tools will improve our understanding of the underlying biological processes of interest and allow us to take these limitations into account. Diagnostic test performance adds to the complexity of prevalence measures (Table 2). Additionally, as these diseases vary geographically, the prevalence is characterised, to various extents, by spatial heterogeneity. For example, for STH, sampling multiple villages/schools per implementation unit improves the accuracy in assessing progress towards targets [17]. Furthermore, spatial correlation can be beneficially used to optimise survey designs and improve the accuracy of predictive risk maps [25]. However, geostatistical models for disease prevalence strongly rely on the quality of the underlying data, especially on the reliability of the geographical coordinates of the survey locations [26]. Inaccuracies or incompleteness of this essential information reduces the quality of model outputs.

## Uncertain epidemiology—Learning more

As these diseases are neglected, and often characterised by complicated parasite life cycles, there is limited knowledge on their epidemiology and the population biology of the parasites causing them. Modelling insights remain limited by the lack of epidemiological and field data available [5]. Consequently, modelling assumptions have to be made resulting in uncertainty in model recommendations. There are key areas of uncertainty where epidemiological data are required for improving our understanding of the dynamics and model parameterisation, in order to improve the robustness of model insights (Table 3 and Fig 1). Although some parameters may never be estimable, there may be testable hypotheses which could inform our understanding of epidemiology.

The persistence of transmission when infection levels have been reduced through interventions is crucially dependent on heterogeneities in exposure, immunological processes, parasite aggregation, and ultimately transmission. These are very difficult to measure, even in epidemiological studies, but may be essential for achieving the long-term goals of NTD programmes. For vector-borne diseases, such as onchocerciasis and visceral leishmaniasis, human/vector mixing patterns play a role in local transmission dynamics. Hence, data on these patterns can reveal the degree of spatial clustering, assortative (nonhomogeneous) mixing and exposure heterogeneity allowing for improved prediction of village-level incidence and guidelines on spatially targeted interventions [14,15,22,27]. Additionally, for visceral leishmaniasis, data on immune responses and infection combined with presence or absence of symptoms can inform the duration of immunity and identify markers for infection [23,28]. Note that we focus on visceral leishmaniasis in the Indian subcontinent as it is believed to be entirely anthroponotic only there (i.e., humans are the only reservoir of infection) [22].

Water, sanitation, and hygiene (WASH) interventions have played a role across many of the NTDs. However, the value of WASH has been difficult to analyse with reviews based on current evidence showing contrasting effects [29–31]. To better understand and predict the added value of WASH, detailed data on WASH-related behaviour are required, although this could be difficult to collect [18] (Table 3).

### Better data but at what cost?

**Fig 2:**
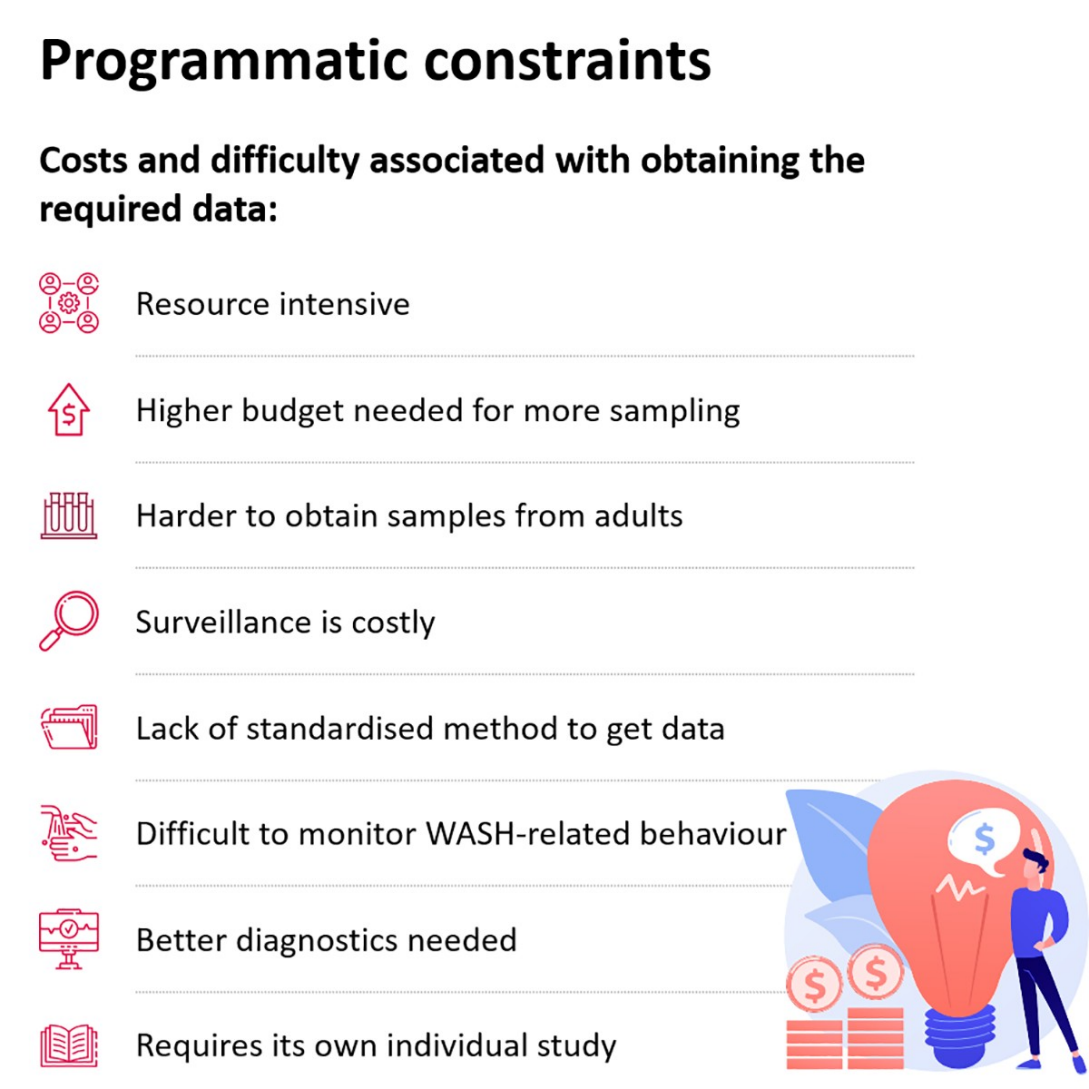
Programmatic constraints associated with obtaining the required M&E and epidemiological data. M&E, monitoring and evaluation; WASH, water, sanitation, and hygiene.

It is important to take into account that although there are great benefits to better data, data collection is typically limited due to various financial and programmatic constraints. Key constraints associated with obtaining data are summarised in Tables 2 and 3 and Fig 2.

Although it is likely to be more costly to collect the required data, this may be more cost-effective in the long term as it will allow for more effective decision-making. Hence, rather than a cost, this could be viewed as an investment. As an example for schistosomiasis, new diagnostic techniques may potentially have a higher cost per test, but this may be outweighed by the long-term programmatic benefits, including being able to detect elimination and resurgence [32]. Furthermore, given the similarities of data needs for these diseases, integration of data collection activities across multiple NTDs could potentially reduce the total costs.

### Data curation, integration, and availability

There are a variety of challenges surrounding the quality of current data, for example, data collected on paper that requires manual entry into databases can increase the risk of errors and be time-consuming. Other challenges include partial reporting whereby only a portion or summary of the data collected is made available, and the absence of standardisation and consistency of reporting both within and between countries at different time points can make the data integration process difficult often resulting in a loss of data. Hence, better data refers not only to collecting a greater quantity of data but also to improving the quality of the data and data reporting protocols. For the NTD Modelling Consortium and for the wider scientific community, data curation, integration, and availability are key. Standardising and curating data and having it available publicly would ensure that it can be utilised by the scientific community. Electronic data collection tools are paving the way forward for addressing some of these challenges [33–36]. Alongside this, the Findability, Accessibility, Interoperability, and Reusability (FAIR) data principles have been designed to improve scientific data management and stewardship [37]. Publishing the models and outputs in a reproducible way is also important for driving forward progress on NTDs.

## Conclusions

Better M&E and epidemiological data will improve our understanding of these NTDs by leading to more informed parameter values, validated model structures, and reduced uncertainty, thereby improving the reliability of assessments of intervention programmes and modelling recommendations for tailored interventions. On the one hand, more accurate models may give us greater confidence in whether the goal of an intervention strategy will be met. On the other, they might allow us to better assess the robustness of M&E strategies, which aim to verify whether a goal has been met, after an intervention has been implemented.

Further work is needed to encourage opportunities for the integration of data collection activities across the NTDs and where possible, a wider spectrum of diseases. Additionally, once NTD programmes are able to resume following the current disruption due to COVID-19, potential synergies between the COVID-19 control efforts and NTD programmes will be important to consider [10,11,38]. Moving forward, as transmission declines and programmes become more tailored, such opportunities will be important as data needs will continue to grow.

## References

[1] World Health Organization. Accelerating work to overcome the global impact of neglected tropical diseases: a roadmap for implementation. 2012 [cited 2021 Jan 30]. Available from: https://www.who.int/neglected_diseases/NTD_RoadMap_2012_Fullversion.pdf

[2] World Health Organization. Ending the neglect to attain the Sustainable Development Goals: a road map for neglected tropical diseases 2021–2030. 2020 [cited 2021 Jan 30]. Available from: https://www.who.int/neglected_diseases/resources/who-ucn-ntd-2020.01/en/

[3] Malecela MN. Reflections on the decade of the neglected tropical diseases. Int Health. 2019. 10.1093/inthealth/ihz048 31529110

[4] Hollingsworth TD, Adams ER, Anderson RM, Atkins K, Bartsch S, Basáñez MG, et al. Quantitative analyses and modelling to support achievement of the 2020 goals for nine neglected tropical diseases. Parasit Vectors. 2015. 10.1186/s13071-015-1235-1 26652272 PMC4674954

[5] Hollingsworth TD. Counting Down the 2020 Goals for 9 Neglected Tropical Diseases: What Have We Learned From Quantitative Analysis and Transmission Modeling? Clin Infect Dis. 2018. 10.1093/cid/ciy284 29860293 PMC5982793

[6] Hollingsworth TD, Medley GF. Learning from multi-model comparisons: Collaboration leads to insights, but limitations remain. Epidemics. 2017. 10.1016/j.epidem.2017.02.014 28279450

[7] Gates Open Research. 2030 goals for neglected tropical diseases. 2020 [cited 2020 June 11]. Available from: https://gatesopenresearch.org/collections/ntd

[8] World Health Organization. Modelling study widens viewpoints for new roadmap for neglected tropical diseases. 2019 [cited 2020 June 11]. Available from: https://www.who.int/neglected_diseases/news/modelling-study-widens-viewpoints-for-new-modelling-NTD-roadmap/en/

[9] PLOS Collections. NTD Modelling Consortium: Insights on data needs. 2019 [cited 2020 May 28]. Available from: https://collections.plos.org/modellingfordata

[10] World Health Organization. COVID-19: WHO issues interim guidance for implementation of NTD programmes. 2020 [cited 2020 June 11]. Available from: https://www.who.int/neglected_diseases/news/COVID19-WHO-interim-guidance-implementation-NTD-programmes/en/

[11] Toor J, Adams ER, Aliee M, Amoah B, Anderson RM, Ayabina D, et al. Predicted impact of COVID-19 on neglected tropical disease programmes and the opportunity for innovation. Clin Infect Dis. 2020. 10.1093/cid/ciaa933 32984870 PMC7543306

[12] Castano MS, Ndeffo-Mbah ML, Rock KS, Palmer C, Knock E, Miaka EM, et al. Assessing the impact of aggregating disease stage data in model predictions of human African trypanosomiasis transmission and control activities in Bandundu province (DRC). PLoS Negl Trop Dis. 2020. 10.1371/journal.pntd.0007976 31961872 PMC6994134

[13] Michael E, Sharma S, Smith ME, Touloupou P, Giardina F, Prada JM, et al. Quantifying the value of surveillance data for improving model predictions of lymphatic filariasis elimination. PLoS Negl Trop Dis. 2018. 10.1371/journal.pntd.0006674 30296266 PMC6175292

[14] de Vos AS, Stolk WA, de Vlas SJ, Coffeng LE. The effect of assortative mixing on stability of low helminth transmission levels and on the impact of mass drug administration: Model explorations for onchocerciasis. PLoS Negl Trop Dis. 2018. 10.1371/journal.pntd.0006624 30296264 PMC6175282

[15] Hamley JID, Milton P, Walker M, Basáñez MG. Modelling exposure heterogeneity and density dependence in onchocerciasis using a novel individual-based transmission model, EPIONCHO-IBM: Implications for elimination and data needs. PLoS Negl Trop Dis. 2019. 10.1371/journal.pntd.0007557 31805049 PMC7006940

[16] Toor J, Turner HC, Truscott JE, Werkman M, Phillips AE, Alsallaq R, et al. The design of schistosomiasis monitoring and evaluation programmes: The importance of collecting adult data to inform treatment strategies for Schistosoma mansoni. PLoS Negl Trop Dis. 2018. 10.1371/journal.pntd.0006717 30296257 PMC6175503

[17] Giardina F, Coffeng LE, Farrell SH, Vegvari C, Werkman M, Truscott JE, et al. Sampling strategies for monitoring and evaluation of morbidity targets for soil-transmitted helminths. PLoS Negl Trop Dis. 2019. 10.1371/journal.pntd.0007514 31242194 PMC6615707

[18] Coffeng LE, Nery SV, Gray DJ, Bakker R, de Vlas SJ, Clements ACA. Predicted short and long-term impact of deworming and water, hygiene, and sanitation on transmission of soil-transmitted helminths. PLoS Negl Trop Dis. 2018. 10.1371/journal.pntd.0006758 30522129 PMC6283645

[19] Pinsent A, Hollingsworth TD. Optimising sampling regimes and data collection to inform surveillance for trachoma control. PLoS Negl Trop Dis. 2018. 10.1371/journal.pntd.0006531 30307939 PMC6181273

[20] Lietman TM, Deiner MS, Oldenburg CE, Nash SD, Keenan JD, Porco TC. Identifying a sufficient core group for trachoma transmission. PLoS Negl Trop Dis. 2018. 10.1371/journal.pntd.0006478 30296259 PMC6175502

[21] Chapman LAC, Morgan ALK, Adams ER, Bern C, Medley GF, Hollingsworth TD. Age trends in asymptomatic and symptomatic Leishmania donovani infection in the Indian subcontinent: A review and analysis of data from diagnostic and epidemiological studies. PLoS Negl Trop Dis. 2018. 10.1371/journal.pntd.0006803 30521526 PMC6283524

[22] Chapman LAC, Jewell CP, Spencer SEF, Pellis L, Datta S, Chowdhury R, et al. The role of case proximity in transmission of visceral leishmaniasis in a highly endemic village in Bangladesh. PLoS Negl Trop Dis. 2018. 10.1371/journal.pntd.0006453 30296295 PMC6175508

[23] Bulstra CA, Le Rutte EA, Malaviya P, Hasker EC, Coffeng LE, Picado A, et al. Visceral leishmaniasis: Spatiotemporal heterogeneity and drivers underlying the hotspots in Muzaffarpur, Bihar, India. PLoS Negl Trop Dis. 2018. 10.1371/journal.pntd.0006888 30521529 PMC6283467

[24] Singh OP, Tiwary P, Kushwaha AK, Singh SK, Singh DK, Lawyer P, et al. Xenodiagnosis to evaluate the infectiousness of humans to sandflies in an area endemic for visceral leishmaniasis in Bihar, India: a transmission-dynamics study. Lancet. 2021. 10.1016/S2666-5247(20)30166–X PMC7869864 33615281

[25] Fronterre C, Amoah B, Giorgi E, Stanton MC, Diggle PJ. Design and Analysis of Elimination Surveys for Neglected Tropical Diseases. J Infect Dis. 2020. 10.1093/infdis/jiz554 31930383 PMC7289555

[26] Jacquez GM. A research agenda: does geocoding positional error matter in health GIS studies? Spat Spatiotemporal Epidemiol. 2012. 10.1016/j.sste.2012.02.002 22469487 PMC3319654

[27] Chapman LAC, Spencer SEF, Pollington TM, Jewell CP, Mondal D, Alvar J, et al. Inferring transmission trees to guide targeting of interventions against visceral leishmaniasis and post–kala-azar dermal leishmaniasis. Proc Natl Acad Sci U S A. 2020. 10.1073/pnas.2002731117 32973088 PMC7568327

[28] Le Rutte EA, Zijlstra EE, de Vlas SJ. Post-Kala-Azar Dermal Leishmaniasis as a Reservoir for Visceral Leishmaniasis Transmission. Trends Parasitol. 2019. 10.1016/j.pt.2019.06.007 31266711 PMC6667731

[29] Stocks ME, Ogden S, Haddad D, Addiss DG, McGuire C, Freeman MC. Effect of water, sanitation, and hygiene on the prevention of trachoma: a systematic review and meta-analysis. PLoS Med. 2014. 10.1371/journal.pmed.1001605 24586120 PMC3934994

[30] Vaz Nery S, Pickering AJ, Abate E, Asmare A, Barrett L, Benjamin-Chung J, et al. The role of water, sanitation and hygiene interventions in reducing soil-transmitted helminths: interpreting the evidence and identifying next steps. Parasit Vectors. 2019. 10.1186/s13071-019-3532-6 31138266 PMC6540378

[31] Ejere HOD, Alhassan MB, Rabiu M. Face washing promotion for preventing active trachoma. Cochrane Database Syst Rev. 2015. 10.1002/14651858.CD003659.pub4 25697765 PMC4441394

[32] Turner HC, Bettis AA, Dunn JC, Whitton JM, Hollingsworth TD, Fleming FM, et al. Economic Considerations for Moving beyond the Kato-Katz Technique for Diagnosing Intestinal Parasites As We Move Towards Elimination. Trends Parasitol. 2017. 10.1016/j.pt.2017.01.007 28187989 PMC5446322

[33] World Health Organization Expanded Special Project for Elimination of Neglected Tropical Diseases. ESPEN Collect. 2021 [cited 2021 Jan 30]. Available from: https://espen.afro.who.int/tools-resources/espen-collect

[34] Open Data Kit. Open Data Kit. 2020 [cited 2021 Jan 30]. Available from: https://opendatakit.org/

[35] Epicollect5. Free and easy-to-use mobile data-gathering platform. 2021 [cited 2021 Jan 30]. Available from: https://five.epicollect.net/

[36] Tropical Data. 2021 [cited 2021 Mar 22]. Available from: https://www.tropicaldata.org

[37] Wilkinson M, Dumontier M, Aalbersberg IJ, Appleton G, Axton M, Baak A, et al. The FAIR Guiding Principles for scientific data management and stewardship. Sci Data. 2016. 10.1038/sdata.2016.18 26978244 PMC4792175

[38] Ehrenberg JP, Zhou XN, Fontes G, Rocha EMM, Tanner M, Utzinger J. Strategies supporting the prevention and control of neglected tropical diseases during and beyond the COVID-19 pandemic. Infect Dis Poverty. 2020. 10.1186/s40249-020-00701-7 32646512 PMC7347419

